# PTI‐ETI synergistic signal mechanisms in plant immunity

**DOI:** 10.1111/pbi.14332

**Published:** 2024-03-12

**Authors:** Xiao‐Qian Yu, Hao‐Qiang Niu, Chao Liu, Hou‐Ling Wang, Weilun Yin, Xinli Xia

**Affiliations:** ^1^ State Key Laboratory of Tree Genetics and Breeding College of Biological Sciences and Technology, College of Biological Sciences and Technology, Beijing Forestry University Beijing China

**Keywords:** PTI, ETI, PRRs, NLRs, ROS, Ca^2+^ influx

## Abstract

Plants face a relentless onslaught from a diverse array of pathogens in their natural environment, to which they have evolved a myriad of strategies that unfold across various temporal scales. Cell surface pattern recognition receptors (PRRs) detect conserved elicitors from pathogens or endogenous molecules released during pathogen invasion, initiating the first line of defence in plants, known as pattern‐triggered immunity (PTI), which imparts a baseline level of disease resistance. Inside host cells, pathogen effectors are sensed by the nucleotide‐binding/leucine‐rich repeat (NLR) receptors, which then activate the second line of defence: effector‐triggered immunity (ETI), offering a more potent and enduring defence mechanism. Moreover, PTI and ETI collaborate synergistically to bolster disease resistance and collectively trigger a cascade of downstream defence responses. This article provides a comprehensive review of plant defence responses, offering an overview of the stepwise activation of plant immunity and the interactions between PTI‐ETI synergistic signal transduction.

## Introduction

The intricate dance of life is often overshadowed by the unseen battles that unfold within the plant kingdom, where sessile organisms engage in a constant arms race with their microbial adversaries. As the cornerstone of terrestrial ecosystems, plants have evolved a sophisticated array of defence mechanisms to counteract the relentless onslaught of pathogens. This dynamic interplay between plants and their environment is a testament to the evolutionary prowess of both parties, with plants developing a multi‐tiered immune system that includes both PTI and ETI (Boller and Felix, 2009; Meng and Zhang, 2013). PTI represents the first line of defence, where plant cell surface receptors recognize conserved microbial patterns, initiating a rapid response to potential threats (Rao *et al*., 2018; Sun *et al*., 2020). ETI, on the other hand, is a more specialized and robust defence mechanism that targets specific pathogen effectors, often resulting in a localized hypersensitive response to halt infection (Boller and Felix, 2009; Prautsch *et al*., 2023). In this dynamic interplay, pathogens may evolve to breach the first line of defence, entering plant cells to produce effectors that suppress the primary immune response, enhance virulence and interfere with PTI, thereby facilitating infection (Albert *et al*., 2015, 2020). Plants counter this by employing a secondary defence strategy called ETI, which involves a family of intracellular polymorphic NLR receptors (Aarts *et al*., 1998). These receptors are activated by the specific recognition of pathogen effectors, triggering a rapid defence response that restores and amplifies the PTI transcriptional programme, often leading to localized cell death at the infection site, induction of systemic acquired resistance and immune stimulation at non‐infected sites (Jones and Dangl, 2006). Typically, PTI and ETI recognize distinct plant pathogens and are considered functionally and mechanistically independent branches of the plant immune system (Jones and Dangl, 2006). However, recent research has revealed that immune components in PTI and ETI have coevolved across different plant species, blurring the distinction between the two (Zhang *et al*., 2023b). They are now recognized as interdependent components of a unified system. The qualitatively similar defence outputs of the PTI and ETI pathways suggest that immune signalling pathways converge upstream of nuclear events and that pathways initiated in different subcellular compartments collaborate to provide robust immune response.

The interplay between PTI and ETI is a complex and highly regulated process that involves a cascade of signalling events, including the generation of reactive oxygen species (ROS) and calcium ion fluxes, which serve as critical second messengers in the plant's immune response (Atkinson *et al*., 1996; Lin *et al*., 2022a). These signalling pathways are intricately linked, with PTI often priming the plant for a more potent ETI response (Li *et al*., 2021b). The molecular dialogue between these two branches of the immune system is further modulated by a suite of transcription factors and hormone signalling pathways, which together orchestrate a coordinated defence against a diverse array of pathogens (Jones and Dangl, 2006; Ngou *et al*., 2022a).

Despite the significant advances in our understanding of plant immunity, the precise mechanisms underlying the crosstalk between PTI and ETI, as well as their integration with other plant defence responses, remain areas of active research (Ngou *et al*., 2022b). This review aims to provide a comprehensive overview of the current knowledge on the stepwise activation of endogenous immunity in plants, focusing on the recognition of extracellular and intracellular effectors by PRRs and NLRs respectively. This review summarizes the stepwise activation of plant endogenous immunity, with a focus on the recognition of extracellular and intracellular effectors by PRRs and NLRs, emphasizing the complex interplay between these pathways and their contributions to the overall plant immune response.

## Stepwise activation of endogenous immunity in plants

**Figure 1:**
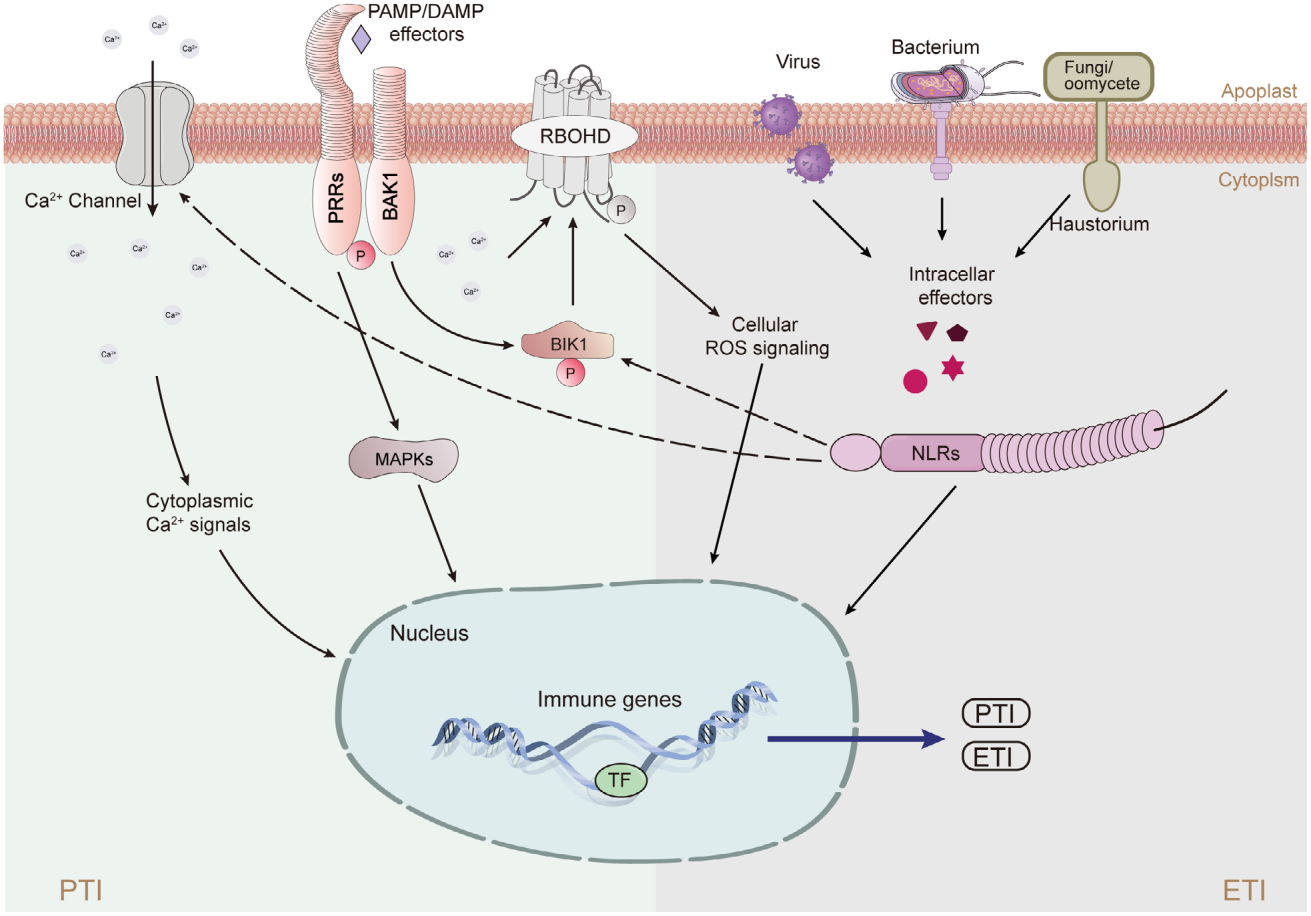
An overview of plant immune signalling networks. Pathogen‐associated molecular patterns (PAMPs) and damage‐associated molecular patterns (DAMPs) are recognized by pathogen recognition receptors (PRRs) on the cell surface, such as FLS2 and BAK1, leading to PAMP‐triggered immunity (PTI), activating the MAPK cascade and intracellular Receptor‐like cytoplasmic kinases (RLCKs) phosphorylate RBOHD and calcium ion channels GLRs, CNGCs, OSCA, activate ROS generation and increase cytoplasmic calcium ion concentration (Atkinson *et al*., 1996; Lin *et al*., 2022a, 2022b; Sun *et al*., 2013; Zhang *et al*., 2023a). The effectors produced by bacterium, virus, fungi and oomycetes entering the cell are recognized by NOD‐like receptors (NLRs) to initiate effector‐triggered immune ETI (Dodds and Rathjen, 2010; Jin *et al*., 2023). Arrows and end‐blocked lines indicate positive and negative regulation respectively. The letter P indicates phosphorylation. The TF indicates transcription factor.

The dynamic interactions between plants and microorganisms in their surroundings have shaped plants with adaptable and intricate immune signalling networks. Through the process of natural selection, the host has evolved a robust, two‐tiered innate immune system in plants that is highly conserved, enabling them to recognize and respond to the signals from a multitude of pathogenic microorganisms (Figure 1) (Dodds and Rathjen, 2010).

### Recognition of MAMPs/PAMPs by PRRs

**Figure 2:**
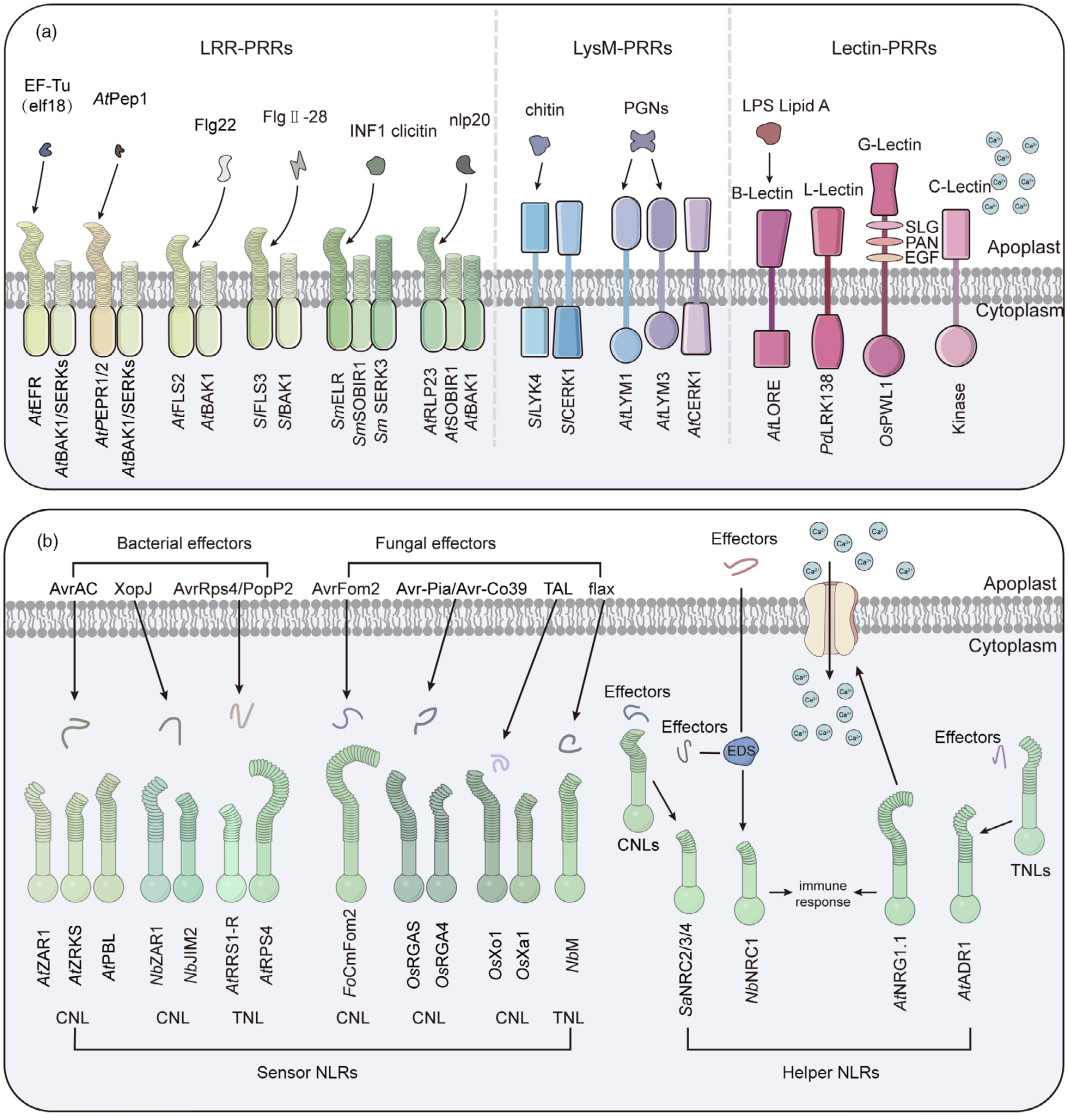
Mediation of immune signalling by PRRs and NLRs. (a) LRR‐PRRs, including LRR‐RKs and LRR‐RPs, bind proteins and peptides via their LRR domain. LRR‐RK EFR and PEPR1/PEPR2 recognize elf18 and AtPep1, respectively, and form heterodimers with BAK1 or SERKs co‐receptors (Bohm *et al*., 2014a; Boller and Felix, 2009; Lacombe *et al*., 2010; Zipfel, 2014). FLS2 and FLS3 specifically bind BAK1 and recognize peptides (Felix *et al*., 1999; Hind *et al*., 2016; Sun *et al*., 2013). The LRR domain of ELR has a strong affinity with INF1 elicitin and recruits SOBIR1 and SERK3 as assistants (Domazakis *et al*., 2018). RLP23 specifically recognizes the nlp20, forming a complex with SOBIR1 and BAK1 (Albert *et al*., 2015). LysM‐RPs and LysM‐RKs detect fungal chitin and bacterial PGN, with LYK4 showing high chitin affinity and inducing LYK4‐CERK1 complex formation (Ai *et al*., 2023; Gust *et al*., 2012). Three LysM domain proteins, LYM1, LYM3 and CERK1, sense bacterial PGNs (Willmann *et al*., 2011). LecRLKs, categorized by ECD, include B‐type with a bulb‐type lectin domain for lipid A detection, L‐type with a legume lectin‐like domain, G‐type with α‐mannose‐binding, SLG, PAN, and/or EGF domains, and C‐type with a calcium‐dependent lectin domain (Cambi *et al*., 2005; Ma *et al*., 2018a; Ranf *et al*., 2015; Sun *et al*., 2020; Xu *et al*., 2023). (b) Plant NLRs are categorized as sensor NLRs and helper NLRs based on function, and as TNLs and CNLs based on N‐terminal structure. CNL ZAR1 recognizes AvrAC and XopJ via ZRKS, PBL and JIM2 respectively (Bi *et al*., 2021; Schultink *et al*., 2019; Wang *et al*., 2015b), while CNL Xo1 and Xa1 act as TAL receptors (Read *et al*., 2020). TNL RRS1 pairs with RPS4 to detect AvrRps4 and PopP2, with RRS1 as a sensor and RPS4 as a signal NLR (Le Roux *et al*., 2015; Ma *et al*., 2018b; Newman *et al*., 2019); fungal effector AvrFom2 is recognized by CNL CmFom2, and flax by TNL M (Catanzariti *et al*., 2010; Schmidt *et al*., 2016). CNL RGA4 and RGA5 heterodimers bind Avr‐Pia and Avr‐Co39 (Vo *et al*., 2019; Wu *et al*., 2015; Zhang *et al*., 2015). Helper NLRs, NRG1.1, transduce immune signals from NLRs and regulate calcium ion influx in conjunction with ADR1 (Jacon *et al*., 2021; Peart *et al*., 2005; Prautsch *et al*., 2023). NRC1, another helper NLR, is activated by effector signals on the cell surface and intracellularly, downstream of EDS, to initiate immune defence (Gabriels *et al*., 2007; Sueldo *et al*., 2015). NRC2/3/4 facilitate CNL effector recognition for defence activation (Ahn *et al*., 2023; Lin *et al*., 2022b; Witek *et al*., 2021). Arrows indicate positive regulation.

The initial layer of defence is composed of cell surface PRRs, which recruit co‐receptors via their extracellular domain to detect the conserved features of invading pathogens, encompassing PAMPs or host‐derived DAMPs, thereby activating the pattern‐triggered immune (PTI) (Figures 1 and 2a) (Wang *et al*., 2022b). Plant PRRs encompassing receptor‐like kinases (RLKs) and receptor‐like proteins (RLPs). RLKs are characterized by a ligand‐binding ectodomain (ECD), a single transmembrane domain and an intracellular kinase structure domain; in contrast, RLPs lack the intracellular kinase domain and are characterized by a short cytoplasmic tail (Boutrot and Zipfel, 2017). The functional domains or motifs within ECDs can recognize a diverse array of ligands, including steroids, polypeptides, polysaccharides, lipopolysaccharides and effectors released by pathogenic bacteria, as well as host endogenous molecules, which are identified as red flags that enhance plant disease resistance (Tang *et al*., 2017). Based on ECDs, known PRRs can be categorized into leucine‐rich repeat (LRR)‐containing PRRs, lysine motifs (LysM)‐containing PRRs, lectin domain‐containing PRRs and epidermal growth factor (EGF)‐like domain (Figure 2a) (Couto and Zipfel, 2016; Tang *et al*., 2017).

Based on the properties of the ligand‐binding ectodomain, LRR‐containing PRRs preferentially bind proteins or peptides, such as the LRR‐RLKs FLS2 and FLS3, which play a pivotal role in the recognition of flg22 and flgII‐28, peptides derived from bacterial flagellin (Felix *et al*., 1999; Hind *et al*., 2016). In the model plant Arabidopsis, BAK1 acts as a co‐receptor for FLS2, reciprocally activating the immune response upon recognize the C‐terminus of flg22 (Sun *et al*., 2013). LRR‐RLKs are known to detect peptides, and certain LRR‐RLPs have ability to recognize proteinaceous patterns. The Arabidopsis LRR‐RK EFR directly recognizes the N‐acetylated epitope elf18, which is defined by the first 18 amino acids of the bacterial elongation factor Tu (EF‐Tu), and this recognition capability has been confirmed in plants of the *Brassicaceae* and *Solanaceae* families (Boller and Felix, 2009; Lacombe *et al*., 2010). AtPEPR1/AtPEPR2 recognizes the plant‐derived AtPep1, similar to AtERF, and forms heterodimeric complexes with different LRR‐RK‐type co‐receptors BAK1 or SERKs (SOMATIC EMBRYOGENESIS RECEPTOR KINASE) (Bohm *et al*., 2014a; Zipfel, 2014). A notable example is the ELICITIN RESPONSE protein (ELR) in *Solanum microdontum*, which can identify INF1 elicitin from *Phytophthora infestans*. ELR and SUPPRESSOR OF BIR1‐1 (SOBIR1) to form a constitutively ELR‐SOBIR1 complex that is activated by INF1 elicitin, subsequently recruiting SERK3 (SOMATIC EMBRYOGENESIS RECEPTOR KINASE 3) to initiate downstream signalling cascades (Domazakis *et al*., 2018). In Arabidopsis, the LRR‐RP RLP23 detects necrosis and ethylene‐inducing peptide 1‐like proteins (NLPs) produced by both prokaryotes and eukaryotes organisms. It specifically binds a conserved 20 amino acid fragment known as nlp20. RLP23 then engages two other LRR‐RKs, SOBIR1 (Suppressor of Brassinosteroid insensitive 1 (BRI1)‐associated kinase (BAK1)‐interacting receptor kinase 1) and BAK1, to form a tripartite complex that mediates immune activation (Albert *et al*., 2015).

LysM‐RPs and LysM‐RKs primarily recognize fungal chitin and bacterial peptidoglycan (PGN) (Gust *et al*., 2012). Lysin motif receptor kinases (LysM‐RKs) are characterized by their extracellular LysM and intracellular Ser/Thr kinase domains. The extracellular domain (ECD) of the tomato LysM‐RK4 (SILYK4) exhibits a higher binding affinity for chitin, which subsequently leads to the formation of a complex between SILYK4‐SICERK1 (chitin elicitor receptor kinase 1), thereby enhancing the resistance to disease (Ai *et al*., 2023). Previous research has identified LysM receptor‐like kinase 1/chitin elicitor receptor kinase1 (LysM RLK1/CERK1) as a pivotal player in the chitin response mechanism in Arabidopsis thaliana (Iizasa *et al*., 2010). The three distinct extracellular LysM domains of CERK1 possess ligand‐binding potential and can directly bind to chitin and partially deacetylated chitosan, without the need for any interacting proteins. This interaction rapidly triggers the phosphorylation of the juxtamembrane and kinase structures of CERK1, which is crucial for defence responses and downstream signalling pathways (Liu *et al*., 2012b; Petutschnig *et al*., 2010). Similar findings have been reported in rice (Shimizu *et al*., 2010). Moreover, two homologous LysM motif‐containing proteins, LYP4 and LYP6, have been identified in rice, functioning as dual‐purpose PRRs that detect bacterial peptidoglycan and fungal chitin (Liu *et al*., 2012a). In addition, a receptor‐independent PGN recognition system has been discovered in Arabidopsis, involving three LysM domain proteins, LYM1, LYM3 and CERK1, which sense bacterial PGNs (Willmann *et al*., 2011).

Lectin‐RLKs are integral to the plant defence mechanisms. They are categorized into four distinct types based on the diversity of their lectin domains: B‐type, L‐type, G‐type and C‐type (Sun *et al*., 2020). The bulb‐type (B‐type) lectin, exemplified by the S‐domain receptor‐like kinase LORE (SD1‐29) in Arabidopsis, primarily detects lipid A of lipopolysaccharide (LPS), playing a pivotal role in LPS sensing and bolstering plant resistance to bacteria (Ranf *et al*., 2015). The overexpression of *PbLRK138*, a representative of L‐type LecRLK, has been shown to induce cell death and stimulate the expression of defence‐related gene in tobacco leaves (Ma *et al*., 2018a,b). G‐type LecRLKs, such as PWL1, demonstrate enhanced disease resistance following functional mutations (Xu *et al*., 2023). C‐type LecRLKs, which bind carbohydrate structures in a Ca^2+^‐dependent manner to mediate innate immune responses in mammals, are less explored in plant immunity (Cambi *et al*., 2005).

Notably, RLKs and RLPs diverge in their co‐receptor recruitment mechanisms, which is dictated by the presence or absence of an intracellular kinase domain for signal transduction. RLKs, upon ligand recognition, are triggered to homodimerize or heterodimerize with co‐receptors, typically somatic embryogenesis RLKs (SERKs) or BAK1 (Chinchilla *et al*., 2009). In contrast, RLPs, lacking an intracellular kinase domain, cannot independently activate PRRs and transduce signals by binding to a signal co‐receptor. Instead, RLP initially interacts constitutively with SOBIR1 to form a heterodimer and subsequently recruits BAK1 or SERK family members to engage in cytoplasmic signal transduction. However, the precise structural mechanism underpinning this process remains elusive (Albert *et al*., 2015). For instance, the LRR‐RLP response to XEG1 (RXEG1) serves as a receptor for the effector XEG1 and its homologues from the pathogen *Phytophthora sojae*. This interaction inhibits the xyloglucanase activity and virulence of XEG1 (Sun *et al*., 2022; Wang *et al*., 2018b). It has been observed that RXEG1 interacts with XEG1, modulating cell death in *N*. *benthamiana* induced by XEG1 and its homologues (Sun *et al*., 2022). The recognition of XEG1 facilitates the in vivo association of the LRR domain of RXEG1 with the LRR domain of BAK1, further highlighting the complexity of these signalling pathways (Sun *et al*., 2022; Wang *et al*., 2018b).

### Rapid phosphorylation of RLCKs

Transphosphorylation between coreceptors and the kinase domain of PRRs (or adapter kinases like SOBIR1) is recognized as the initial intracellular signalling event in response to PAMPs (Albert *et al*., 2020). Receptor‐like cytoplasmic kinases (RLCKs), which lack an extracellular ligand‐binding domain, serve as crucial intermediaries connecting cell‐surface PRRs to both perceive extracellular signals and initiate intracellular responses (Figure S1) (Liang and Zhou, 2018). Arabidopsis alone harbours no fewer than 149 RLCKs, classified into 17 distinct subfamilies (Liang and Zhou, 2018; Shiu *et al*., 2004). BIK1, a member of the RLCK VII subfamily, is instrumental in mediating PTI signal transduction, rapidly phosphorylating residue Thr237 on flg22 in a FLS2‐ and BAK1‐dependent manner. Moreover, BIK1 can also phosphorylate BAK1 and FLS2, indicating the occurrence of a transphosphorylation event between these components (Figure 1) (Lin *et al*., 2013; Lu *et al*., 2010).

RLCKs serve as executors in the downstream signalling cascades of receptor complexes, swiftly phosphorylating downstream proteins to initiate a multitude signalling events (Liang and Zhou, 2018). A notable example involves RLCK VII BIK1, which rapidly phosphorylates SHOU4/4L following flg22 treatment. This phosphorylation events facilitates the dissociation of SHOU4/4L from cellulose synthase 1 (CESA1), underscoring the pivotal role of BIK1‐mediated phosphorylation of SHOU4/4L in both plant immunity and development (Wang *et al*., 2023d). In plant, G proteins, including GPA1, AGB1, AGGs and XLGs in Arabidopsis, form heterotrimers that are integral to plant growth, development, hormone response and defence mechanisms (Bommert *et al*., 2013; Liang *et al*., 2016; Wang *et al*., 2016). Research indicates that XLG2, AGB1 and AGG1/2 are involved in the regulation of PAMP‐triggered immunity by directly interacting with the FLS2‐BIK1 receptor complex. BIK1 is subject to continuous degradation by the 26S proteasome; however, G proteins can mitigate this process, ensuring the host's signal transduction capability. Upon stimulation by flg22, XLG2 dissociates from AGB1 and is phosphorylated by BIK1, which in turn modulates a cascade of downstream reactions, including ROS production (Liang *et al*., 2016). The calcium‐dependent protein kinase CPK28 interacts with and phosphorylates BIK1, contributing to the turnover of BIK1 (Monaghan *et al*., 2014). DAMP Pep1 in Arabidopsis has been identified to induce direct interactions between Pep1 receptor kinases PEPR1/2 and BIK1, as well as PBL1, leading to the phosphorylation of BIK1, which is crucial for ET defence signalling (Liu *et al*., 2013). AtPBL27, a member of RLCK VII, has been demonstrated to promote stomatal closure by phosphorylating the s‐type anion channel AtSLAH3 in response to chitin induction (Liu *et al*., 2019).

The MAPK cascade, which senses endogenous or exogenous stimuli such as DAMPs and P/MAMPs, is a pivotal signalling module that acts downstream of receptors and sensors. It also serves as acts as a central system to phosphorylate downstream target proteins, enabling them to respond to environmental changes (Wang *et al*., 2023c).

In the plant PTI pathway, two primary MAP kinase cascades are involved: one initiated by MAP kinase kinase kinases (MAPKKKs), such as MEKK1, leading to the activation of MAPK4 and MAPK11, and the other by MAPKKK3, MAPKKK5 and YODA, which activate MPK3 and MPK6 (Bi *et al*., 2018; Dong *et al*., 2023; Wang *et al*., 2022c). The activation of mitogen‐activated protein kinases, including MAPK3, MAPK4, MAPK6 and MAPK11, serves as a critical indicator of the immune system response to PRRs, which is essential for establishing disease resistance (Dong *et al*., 2023; Meng and Zhang, 2013). The MEKK1‐MKK1/2‐MPK4 cascade is recognized as a negative regulator of plant innate immunity (Gao *et al*., 2008). The Arabidopsis ASR3, acting as a transcriptional repressor, is activated by the MEKK1‐MKK1/2‐MPK4 cascade to suppress PTI responses (Li *et al*., 2015). Studies indicate that MAPK cascades are activated by PRRs, with RLCK subfamilies VII and XII directly phosphorylating MAPK cascades (Bi *et al*., 2018; Liang and Zhou, 2018; Shi *et al*., 2022). For instance, flg22 and chitin induce FLS2, EFR, CERK1 and PEPRs, leading to MAPKKK3/5 phosphorylation, which subsequently activates MPK3/6 (Bi *et al*., 2018). RLCK VII member PBL19 and CERK1 co‐directly phosphorylate MAPKKK5, activating downstream MAPK6, which in turn phosphorylates MAPKKK5 and induces expression of downstream disease resistance‐related genes (Rao *et al*., 2018). In Arabidopsis, RLCK XII member BSK1 forms a complex with FLS2 to detect flagellin and interacts with MAPKKK5 to phosphorylate it. MAPKKK5, in turn, engages in interactions with multiple MAPK kinases to relay immune signals from the PRR complex to the MAPK cascade (Yan *et al*., 2018). The interaction between RLCKs and MAPKKK5 is facilitated by the involvement of 14–3‐3 proteins, which alleviate the inhibition of RLCKs by MAPKKK5, promote the interaction between RLCKs and the C‐terminus of MAPKKK5, enhance MAP kinase activation and bolster disease resistance (Dong *et al*., 2023). Recent findings indicate that MPK15 can be directly phosphorylated by BSK1, enhancing resistance to powdery mildew fungal infections, with this phosphorylation being further augmented by PAMP elicitation (Shi *et al*., 2022).

Although ETI‐activated immune responses are stronger than those of PTI, the MAPK cascade is a core component for both (Peng *et al*., 2018; Tsuda and Katagiri, 2010). PTI induces a rapid and transient activation of the MAPK cascade, while ETI leads to prolonged and sustained MAPK activity (Tsuda *et al*., 2013; Zipfel, 2009). The effectors AvrRpt2, AvrRpm1 and AvrB target the same host protein RIN4, which interacts with CNL RPM1 and RPS2 and is protected by them, causing sustained MAPK activation (Lang and Colcombet, 2020; Su *et al*., 2018). The sustained activation of MAPK disappears in the knockout lines of genes ALD1 and FMO1 after AvrRpt2 infection, indicating that NLRs are necessary for the sustained activation of the MAPK cascade (Lang and Colcombet, 2020; Lassowskat *et al*., 2014). In Arabidopsis, the MAPK cascade is involved in the negative regulation of PTI and ETI, with the MPK3/MPK6‐WRKY18/40/60 module required for pre‐PTI‐mediated ETI suppression (ETS) (Wang *et al*., 2023a). Arabidopsis mutants *mekk1*, *mkk1;mkk2* and *mpk4* exhibit constitutive immune responses, confirming that the MAPK cascade is protected by NLRs and that the absence of MAPK function leads to ectopic NLR activation (Roux *et al*., 2015).

MYB, ERF, NAC, MYC and WRKY family members function by regulating defence responses following MAPK cascade phosphorylation (Amorim *et al*., 2017; Welch *et al*., 2022; Zhao *et al*., 2021a). WRKY70, WRKY33, WRKY22, WRKY29, WRKY40 and WRKY104 participate in plant defence responses through transcriptional and post‐transcriptional regulation of the MAPK cascade (Asai *et al*., 2002; Hsu *et al*., 2013; Wang *et al*., 2022a; Zhang *et al*., 2022; Zhao *et al*., 2021b; Zhou *et al*., 2022). Phosphorylation of ZmNAC49 by ZmMAPK5 enhances its binding activity to the ZmSOD3 promoter, improving plant oxidative stress tolerance (Xiang *et al*., 2021). MYB transcription factors trigger tissue‐specific immunity through the MKP1‐MPK3/6‐MYB4 signalling cascade (Lin *et al*., 2022a,b). MYB30, MYB44, MYB55 and MYB11 enhance resistance to fungal and bacterial pathogens via the MAPK cascade (Kishi‐Kaboshi *et al*., 2018). ERF6 and ERF104 in Arabidopsis act as substrates of MPK3/6 to modulate plant defence responses (Bethke *et al*., 2009; Meng *et al*., 2013b).

The MAPK cascade is crucial for plant defence hormone regulation. Overexpression of *MKK7* in Arabidopsis boosts salicylic acid and PR gene expression, enhancing resistance (Zhang *et al*., 2007). MAPK4 positively regulates JA signalling, while MYC2, a key JA pathway transcription factor, is negatively regulated by MAPK6 and MKK3 phosphorylation (Petersen *et al*., 2000). CK2 phosphorylates MYC2, affecting JA signalling (Zhu *et al*., 2023). WRKY28 activates *ICS1*, involved in SA biosynthesis (van Verk *et al*., 2011). MPK3 and MPK6 phosphorylation promotes *ACS2* and *ACS6* expression, sustaining ET levels (Figure S2). ERF1A, a JA signalling component, is a substrate for MPK3 and MPK6, and its phosphorylation inhibits ET biosynthesis, induces defence gene expression and enhances resistance with negative feedback regulation (Broekaert *et al*., 2006; Meng and Zhang, 2013).

### Recognition of intracellular effectors by NLRs

Pathogens have evolved complex mechanisms to evade and suppress PTI in their relentless pursuit of survival, secreting effector proteins to facilitate host infection and colonization. Concurrently, plant have evolved NLRs to counter these evasion tactics. These NLRs directly bind to effectors or sense their presence within host cells, triggering the second layer of defence known as ETI (Figures 1 and 2b). Plant NLRs, encoded by Resistance (R) genes, feature a multi‐domain architecture that includes a conserved central nucleotide‐binding domain and a C‐terminal LRR domain. They are classified based on their N‐terminal domain into TIR domains or Frizzled domains, which are further categorized into G10‐type CC‐NLRs (CCG10‐NLRs), RPW8‐type CC‐NLRs (CCR‐NLRs) and Rx‐type coiled‐coil (CC) NLRs (CC‐NLRs) (Kourelis *et al*., 2021). The LRR domain is responsible for the direct or indirect recognition of effectors, the NB‐ARC region possesses ATG binding activity that regulates NLR activation, and the N‐terminal domain is involved in downstream signal transduction following NLR activation (Bi *et al*., 2021; Ngou *et al*., 2022b). Furthermore, NLRs function within gene‐linked receptor pairs or receptor networks, with sensor NLRs (sNLRs) directly recognizing effectors or sensing these transmitted within host cells (Bonardi *et al*., 2011), and helper NLRs (hNLRs) acting as downstream signalling hub for various sNLRs (Jubic *et al*., 2019). Recognition of effectors by plant NLRs typically triggers rapid cell death in the infected cells and surrounding tissues, thus the interactions between effectors, host targets and matching immune receptors are shaped by complex molecular mechanisms and co‐evolutionary dynamics.

Numerous studies have found that bacterial and fungal effectors and molecules are recognized by CNLs and TNLs. NLRs have at least two different signalling branches, CNL appears to signal through NDR1 and TNL appears to signal through EDS1 (Aarts *et al*., 1998). In *N*. *benthamiana*, the CNL NbZAR1 and JIM2 (XopJ4 IMMUNITY 2) have been shown to mediate the recognition of the bacterial effector XopJ4 (Schultink *et al*., 2019). ZAR1 has been shown to sense multiple pathogen effector proteins, acting not only as a sensor for these effectors but also as an executor of signal transduction. In a resting state, ZAR1 interacts with a class of homologous pseudokinases known as ZRKs. Upon pathogen infection, the complex engages a second class of kinases, PBLs, to perceive a variety of effectors, such as AvrAC (Bi *et al*., 2021; Wang *et al*., 2015b). In rice, CNLs OsXo1 and OsXa1 have been identified as receptors for the TAL (TRANSCRIPTION ACTIVATOR‐LIKE) effector from *Xanthomonas oryzae* (Read *et al*., 2020). Additionally, CNLs in *Cucumis melo* have been found to recognize the AvrFom2 effector from *Fusarium oxysporum* (Schmidt *et al*., 2016). Wheat has been observed to utilize multiple CNLs to recognize effectors from *Bacillus graminearum* (Bourras *et al*., 2019; Manser *et al*., 2021; Salcedo *et al*., 2017). Similarly, rice employs multiple CNLs to detect effectors from the rice blast fungus *Magnaporthe oryzae*. RGA5 and RGA4 form functional heterodimers that directly bind to Avr‐Pia and Avr‐Co39, triggering resistance (Vo *et al*., 2019; Wu *et al*., 2015; Zhang *et al*., 2015).

Among TNLs, the most extensively studied are Resistance to Ralstonia solanacearum 1 (RRS1‐R) and Resistance to Pseudomonas syringae 4 (RPS4), which form a TNL pair in Arabidopsis to detect pathogen effectors and initiate defence responses (Le Roux *et al*., 2015; Newman *et al*., 2019). The RRS1‐R‐RPS4 NLR pair, located in the nucleus, recognizes bacterial effectors AvrRps4 and PopP2 through the WRKY transcription factor domain integrated into RRS1‐R. The WRKY domain helps maintain the inactive state of the complex in the absence of effectors, and the interaction of AvrRps4 with the WRKY domain reduces this inhibition (Ma *et al*., 2018a,b). TNLs also play a role in recognizing effectors from fungal pathogens; for instance, the flax TIR‐NB‐LRR protein M directly recognizes effectors from the flax rust pathogen (Catanzariti *et al*., 2010).

Increasing evidence suggests that the recognition of effectors by sNLRs and their conversion to immune activation is dependent on hNLRs. The N REQUIREMENT GENE1 (NRG1), a type of hNLRs, has been identified as playing a role in the downstream signalling pathway of TIR‐NLRs. Alongside ACTIVATED DISEASE RESISTANCE 1 (ADR1), NRG1 oligomerize and localize in the plasma membrane to regulate the calcium ion flow mechanism (Jacon *et al*., 2021; Peart *et al*., 2005; Prautsch *et al*., 2023). NRC family proteins, which include NRG1, have been found to possess multisensor functions in the NLRs of Solanaceae plants (Wu *et al*., 2016). In Arabidopsis ZAR1 and the NRC family, a conserved N‐terminal MADA motif has been identified, which may indicate a similar mechanism for defence activation. Among the hNLRs previously studied, NRC1 has been recognized as a centre signalling hub necessary for both cell surface and intracellular immune receptors to induce cell death (Gabriels *et al*., 2007; Sueldo *et al*., 2015). Further research has identified NRC2a/b and NRC3, but not NRC1, as proteins contributing to Pto‐mediated cell death (Wu *et al*., 2016). In *Solanum americanum*, the CNLs Rpi‐amr3 and Rpi‐amr1 exhibit broad‐spectrum resistance against late blight in potato. Additionally, Rpi‐amr3 and Rpi‐amr1 can recognize the pathogen effectors Avramr3 and Avramr1 from *P*. *infestans* and their homologues, with this recognition process being dependent on NRC2/3/4 and NRC2/3 respectively (Ahn *et al*., 2023; Lin *et al*., 2022a,b; Witek *et al*., 2021).

Furthermore, the effector recognition patterns of plant NLR immune receptors are diverse. For instance, the well‐studied pathogenic *Pseudomonas syringae* in Arabidopsis delivers different type III effectors into the host cell. The type III effectors AvrRpm1 or AvrB are acylated within the host cell, leading to the phosphorylation of RIN4, which then activates the RPM1 that monitors RIN4. RIN4 is also targeted by a third type III effector, AvrRpt2, which is a cysteine protease that undergoes processing within the host cell. The presence of a site within RIN4 that corresponds to the AvrRpt2 cleavage motif leads to the degradation of RIN4, which in turn activates RPS2 (Bialas *et al*., 2018; Kim *et al*., 2005; Mackey *et al*., 2002). Another type of NLR immune receptor activates ETI by mimicking functional proteins to recognize effectors. For example, the intracellular protein kinase Pto found in tomatoes, which resembles the kinase domain of PRRs, directly interacts with type III effector proteins AvrPto or AvrPtoB delivered by *Pseudomonas syringae* (Oh and Martin, 2011; Tang *et al*., 1996; Young *et al*., 2010). In Arabidopsis, the N‐terminal CC domain of the plasma membrane CNL RPS5 interacts with the protein kinase PBS1, maintaining it in an inhibited state. The effector AvrPphB activates RPS5 by cleaving PBS1 (Ade *et al*., 2007). Finally, some NLRs interact with effectors and directly recognize them. For instance, the LRR domain of the TIR‐NLR family member RPP1 in Arabidopsis can specifically recognize the effector ATR1 to trigger immunity (Steinbrenner *et al*., 2012). The flax L5 and L6 TNL allelic forms specifically recognize the effector variant AvrL567 through multiple contact points within the LRR, facilitated by the N‐terminal TIR domain of the receptor (Dodds *et al*., 2006; Ravensdale *et al*., 2012).

## PTI‐ETI crosstalk—The integrative concept of plant immunity

**Figure 3:**
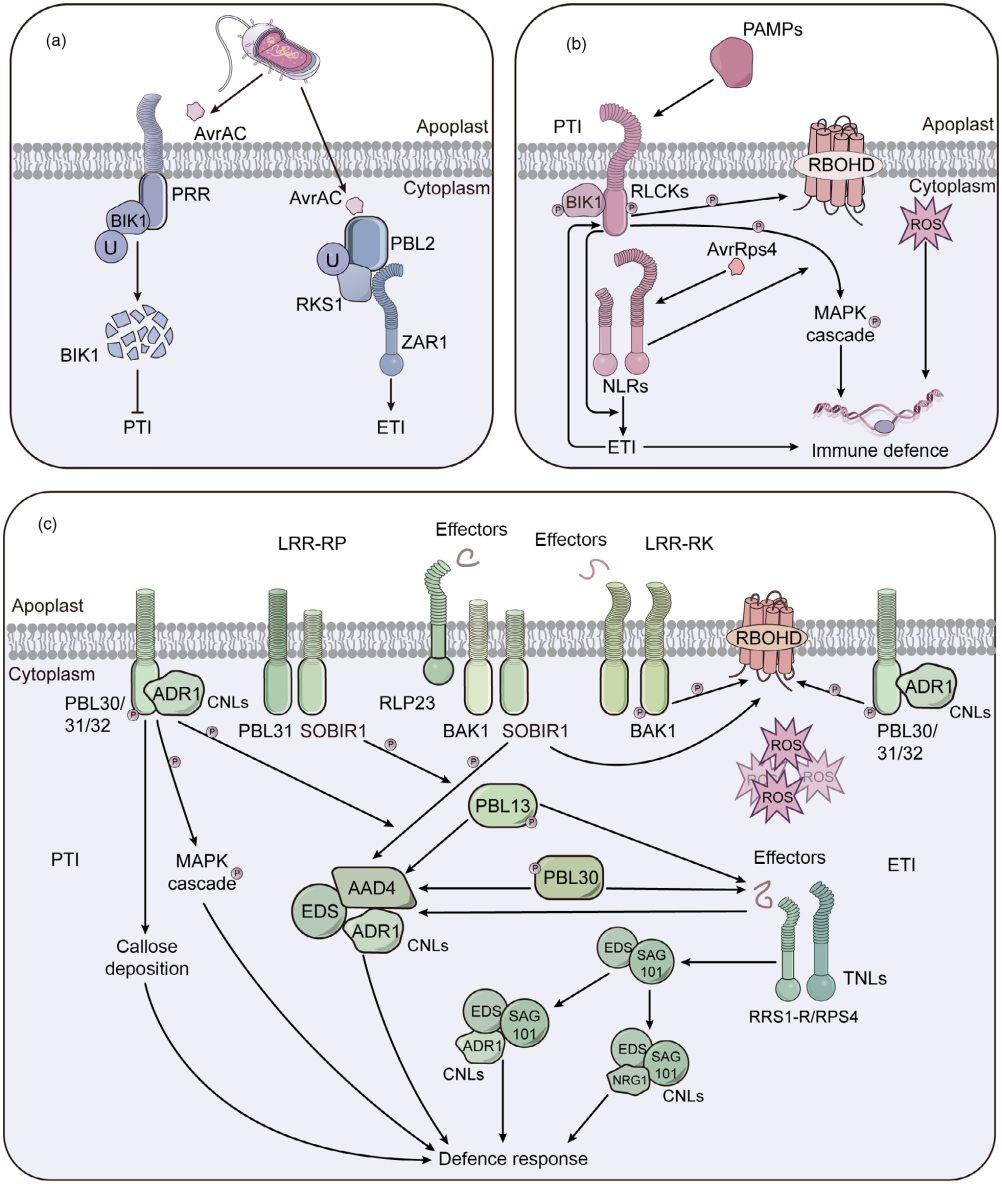
The crosstalk model between PRRs and NLRs mediated immune system. (a) NLRs guard the PRR‐signalling pathway. After the effector AvrAC is recognized by the cell surface receptor PRRs, it mediates BIK1 uridylation and prevents the induction of PTI by degrading BIK1, thereby enhancing the virulence of the effector (Wang *et al*., 2015a). However, since PBL2 is a homologue of BIK1 with a similar structure and is uridylated, RKS1 and ZAR1 detect uridylated PBL and are recruited to activate ETI (Wang *et al*., 2015b). (b) PRRs‐ and NLRs‐signalling pathway synergies amplify each other. Cell surface RLCKs recognize PAMPs to activate PTI and induce ROS production, while intracellular NLRs recognize the effector AvrRps4, which will enhance ROS production, MAPK cascade and promote BIK1 protein accumulation in turn (Ngou *et al*., 2021, 2022a). The cell surface receptor PRRs in the PTI pathway facilitate the recognition of intracellular effectors by NLRs and strengthen plant defence responses (Ngou *et al*., 2021). (c) PRRs‐ and NLRs‐signalling pathways share signalling components. The cell surface recognition receptor LRR‐RP RLP23 recognizes effectors to activate PTI requires an EDS‐PAD4‐ADR1 node (Pruitt *et al*., 2021), the two parties are mainly connected by SOBIR1, and RLCK PBL13/30 contributes to the formation of EDS‐PAD4‐ADR1 node. RLCK PBL30/31/32 interacts with CC_HeLo_‐domain helper NLRs ADR1, facilitating the signal transduction between BAK1‐RLP23‐SOBIR1 and EDS‐PAD4‐ADR1. The ROS generation, MAPK cascade, and callose deposition induced by LRR‐RPs and LRR‐RKs are also assisted by PBL30/31/32‐ADR1, and the activation of TNLs pair RRS1‐S/RPS4 recognition of intracellular effectors requires two EDS modules, which bind differently CC_HeLo_‐domain helper NLRs respectively, NRG1 and ADR1. And TNL pair (RRS1‐S/RPS4) induce the formation of EDS‐SAG101‐ADR1 and EDS‐SAG101‐NRG1 nodes upon activation by effectors (Dongus and Parker, 2021; Lapin *et al*., 2019; Pruitt *et al*., 2021; Sun *et al*., 2021; Tian *et al*., 2021). On the other hand, the EDS‐PAD4‐ADR1 and EDS‐SAG101‐NRG1 node is also required by TNLs to activate the ETI pathway and induce defence responses. It can be seen that the PTI and ETI pathways require a common node to activate downstream defence signals and cause plant immunity. Arrows and end‐blocked lines indicate positive and negative regulation respectively. The letter U indicates uridylation. The letter P indicates phosphorylation.

Extensive research has been conducted on PRR and NLR‐mediated immunity, which, despite being triggered by distinct types of receptors, are increasingly recognized as interdependent components of a unified defence system. NLRs‐mediated immunity was predominantly activated in conjunction with PTI and PTI + ETI. Instances of NLR activation without PTI are relatively rare. The crosstalk between PRRs and NLR‐mediated immune systems typically involves synergistic interactions between ROS, Ca^2+^ and phytohormones, leading to the activation of downstream defence genes through intercellular signalling. This crosstalk enables plants to adapt rapidly to changing conditions while minimizing resource expenditure. In this context, we present three illustrative scenarios (Figure 3).

Several studies have highlighted that PTI serves as the frontline defence, directly targeted by numerous effectors, while NLRs act as a subsequent layer of defence, recognizing multiple effectors to safeguard the PRR signalling pathway. For instance, the *Xanthomonas campestris* effector protein AvrAC/XopAC enhances bacterial virulence, by uridylylating and degrading BIK1 kinase, thereby disrupting basic immunity during host invasion (Figure 3a) (Wang *et al*., 2015a). The receptor‐like cytoplasmic PBL2, a counterpart of BIK1, is similarly uridylated by AvrAC. This modification enables the host to recognize AvrAC and recruits NLR ZAR1 and RLCK XII RKS1 to form a complex to trigger immunity (Wang *et al*., 2015b). Research indicates that the CNLs ZAR1 can directly recognize the pathogen effector HopZ1a, drawing it to the ZAR1 resistance complex via the nonfunctional kinase hopZ‐ETI‐deficient1 (ZED1), which activates ETI (Lewis *et al*., 2013). The Arabidopsis resistance protein RPS5 and the NLR gene AvrPphb Response 1 (PBR1) interact with the serine/threonine protein kinase PBS1 to recognize the bacterial effector AvrPphB (Carter *et al*., 2019). CBP60b, acting as a positive regulator of immunity, promotes *PR* expression upon gain of function and activates EDS1‐PAD4‐initiated NLR signalling upon loss of function, leading to autoimmunity (Li *et al*., 2021a). PICI1 mediates immunity through the synthesis of the ethylene (ET), a plant hormone that is recognized, targeted and degraded by fungal effectors. The NLR PigmR protects PICI1 from degradation, synchronizes ETI with PTI and ensures disease resistance (Zhai *et al*., 2022). Prior studies have established that PTI is essential for effective ETI. The ETI response is significantly compromised in Arabidopsis PRR and PRR co‐receptor mutants, such as *fls2 efr cerk1* and *bak1 bkk1 cerk1* triple mutants. Meanwhile, the receptor‐like cytoplasmic kinase BIK1 activates the NADPH oxidase RBOHD to generate ROS, which serve as an early key signal connecting PRR and NLR‐mediated immunity (Figure 1) (Yuan *et al*., 2021).

Numerous studies underscore the synergistic relationship between PTI and ETI, where they do not function in isolation but rather amplify each other's effects (Figure 3b) (Ngou *et al*., 2022b). Recent research indicates that plant cell surface recognition receptors and intracellular immune receptors work in concert, suggesting a functionally interdependent and mutually reinforcing (Ngou *et al*., 2022c). This concept is illustrated by the ‘Zigzag’ model, which represents the dynamic nature of the plant immune system quantitative output (Jones and Dangl, 2006; Ngou *et al*., 2022a). A pivotal mechanism by which ETI prevents pathogen infection involves enhancing and restoring the turnover of PTI and the activity of pathogen effectors (Ngou *et al*., 2022a). In Arabidopsis, the preactivation of the bacterial effector AvrRps4 augments ROS production induced by flg22, whereas AvrRps4 alone did not trigger ROS production. Furthermore, the preactivation or coactivation of ETI significantly enhances the protein level of BIK1, RBOHD and MPK3 induced by PTI (Ngou *et al*., 2021).

Moreover, mounting evidence suggests that the immune response mediated by NLRs is bolstered by the activation of PRRs. In Arabidopsis, resistance to *P*. *syringae* relies on RRS1/RPS4 and RRS1B/RPS4B during PTI, yet the *rps4rps4b* mutant fails to recognize AvrRps4, a pathogen effector recognized by TIR‐NLRS, highlighting that ETI alone, without the support of PTI, is insufficient for enhanced plant disease resistance (Ngou *et al*., 2021). In tomatoes, the helper NLR SINRC4a associates with the PRR LeEIX2, amplifying the defence responses triggered by EIX (Leibman‐Markus *et al*., 2018). The *slnrc4a* mutant confers broad‐spectrum disease resistance by upregulating PRR expression and volatile products, and recent research indicates that the *slnrc4a/slnrc4b* double mutant exhibits even superior resistance (Leibman‐Markus *et al*., 2023; Pizarro *et al*., 2020). The interplay of negative regulation between PTI and ETI allows plants to sustain a robust immune response. Plants pre‐exposed to flg22 can enhance their resistance when challenged by DC3000 (Pst), a pathogen known to carry effectors that are recognized by both CNLs (C‐terminal NLRs) and TNLs. The regulatory circuit involving Flg22/PIPs‐RLK7‐MPK3/MPK6‐WRKY18/40/60‐prePIPs is implicated in the suppression of ETI during pre‐PTI (PES) (Wang *et al*., 2023a,b,c,d,e).

Additionally, some studies have highlighted the interdependence between the signalling components of PTI and ETI (Figure 3c). Although PTI and ETI are initiated in distinct cellular compartments of the host, they share common pathways for transcriptional activation indicating a potential upstream association of nuclear events (Ngou *et al*., 2022a). For instance, the EDS‐PAD4‐ADR1 signalling node is activated by cell surface RLCPs and some RLCKs, as well as intracellular NLRs receptors, acting as a convergency point for defence signalling pathways to regulate defence responses (Pruitt *et al*., 2021). In Arabidopsis, the bacterial effector avrRps4 activates the receptor pair (RRS1‐S/RPS4), and the ETI response is driven by two distinct EDS modules, EDS‐SAG101 and EDS‐PAD4, which bind to different CC‐domain helper NLRs (RNLs), NRG1 and ADR1 respectively (Dongus and Parker, 2021). The LRR receptor protein RLP23, which triggers PTI, requires the assistance of lipase‐like proteins EDS1, PAD4 and helper NLRs ADR1, with RLCK SOBIR1 acts as a central linker (Pruitt *et al*., 2021; Tian *et al*., 2021). Additionally, SOBIR1 forms a combinatorial complex with RLCK PBL31 for PTI signalling. RLCK PBL30/31/32 and RLP23 in recognizing nlp20 leading to the induction of ROS, callose deposition and MAPK phosphorylation (Bohm *et al*., 2014b; Pruitt *et al*., 2021). RNLs, which activate the ETI pathway, also necessitate the involvement of the EDS‐SAG101‐ADR1 node (Lapin *et al*., 2019; Sun *et al*., 2021). Certain cell surface recognition receptors necessitate the participation of helper NLRs from the NRC family to recognize effectors and trigger hypersensitivity reactions. For example, in *N*. *benthamiana*, NRC3 is essential for the cell surface recognition receptor Cf‐4 to recognize the fungal effector Avr4, leading to cell death (Kourelis *et al*., 2022). The cell surface recognition receptor Ve1 in tomatoes confers resistance to *V*. *dahliae* and *V*. *albo‐atrum*, with EDS and NDR1 as downstream components, and NRC2 and BAK1 are recognized as positive regulators of Ve1 (Fradin *et al*., 2009).

Furthermore, both the PTI and ETI pathways are activated downstream of BAK1 to orchestrate effective plant immunity. The TIR‐NBS‐LRR protein CONSTITUTIVE SHADE AVOIDANCE 1 (CSA1) forms a physical interaction with BAK1‐interacting receptor‐like kinases (BIR3), safeguarding homeostasis of BIR3 and BAK1 and integrating both pattern‐ and effector‐triggered cell death downstream of BAK1. In *bak1‐4* and *bak1‐4 bir3‐2* mutants, CSA1 mediates cell death through the ETI pathway, and in the absence of functional BAK1 and BIR3, the derepression of *CSA1* leads to ETI‐induced cell death (Schulze *et al*., 2022). The endogenous hormone regulation within PTI and ETI also exhibits differences and interactions that are linked to disease susceptibility (ETS). Early infection by *Xanthomonas campestris pv*. *Campestris* (Xcc) leads to the upregulation of *BIK1* expression and accumulation of SA and JA. At later stages, the CNLs *ZAR1* were induced and SA levels rose in vivo, while the increased expression of TNLs *TAO1* correlated with elevated SA and ABA accumulation (Mamun *et al*., 2021).

### ROS and Ca^2+^ signalling in PTI‐ETI synergistic signal transduction

PTI and ETI operate in distinct temporal and spatial domains, necessitating the use of second messengers to serve as ‘language codes’ that convey external signals to the cell interior and mobilize downstream signalling networks (Marcec *et al*., 2019). Growing evidence underscores the significance of ROS and Ca^2+^ signalling in the fine‐tuning of cellular networks within the plant's immune response.

### ROS signalling

**Figure 4:**
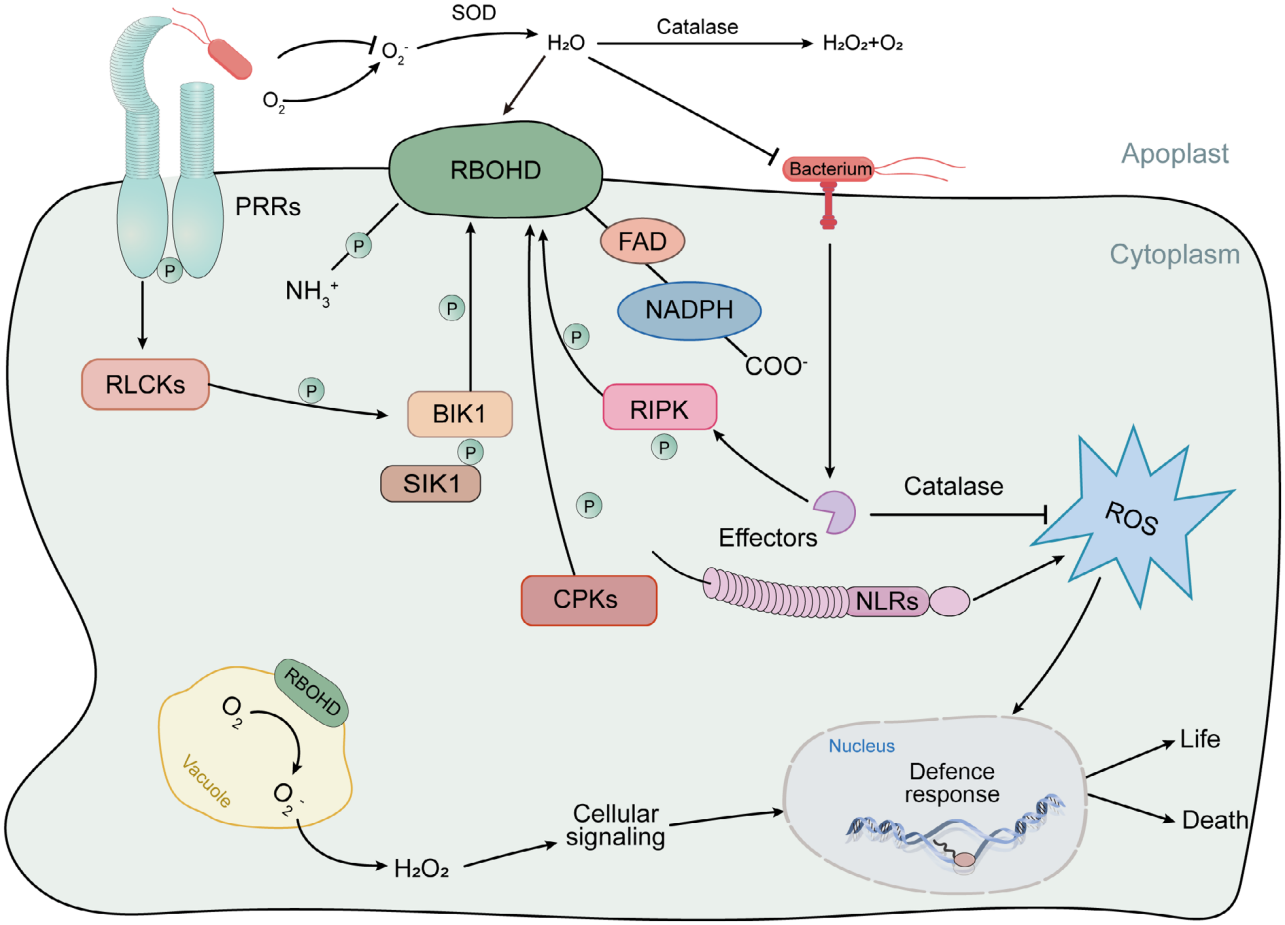
Simplified working model of ROS generation and scavenging in plant immunity. In PTI, PRRs recruit receptor‐like phosphokinases BIK1 to phosphorylate RBOHD and induce ROS generation, extracellular hydrogen peroxide enters the cell interior through reactive oxygen channels and accumulates, activating the primary defence response (Couto and Zipfel, 2016; Loo *et al*., 2022). The Calcium‐dependent protein Kinases CPKs can also phosphorylate RBOHD to promote the production of ROS during the immune process (Dubiella *et al*., 2013; Wang *et al*., 2018a). In ETI, NOD‐like receptor (NLRs) recognizes intracellular effectors to recruit the phosphokinase RIPK to phosphorylate RBOHD to enhance ROS production and strengthen downstream defence responses. Hydrogen peroxide potentially serves as a point of interference for pathogen effectors, and in turn, intracellular pathogen effectors induce catalase to scavenge ROS (Gao *et al*., 2021). ROS acts as an intracellular signal to dynamically activate appropriate defence responses in plants, otherwise leading to cell death. Arrows and end‐blocked lines indicate positive and negative regulation respectively. The letter P indicates phosphorylation.

ROS are chemically reactive molecules containing oxygen, which are continuously produced in plants as toxic byproducts of aerobic metabolism but are also crucial for numerous signalling process (Mittler, 2017). ROS generation plays a pivotal role in plant defence as a conserved immune response, primarily mediated by NADPH oxidases (NOXs), also known as respiratory burst oxidase homologues (RBOHs) (Liu and He, 2016). Both PTI and ETI can lead to the early generation of ROS (Figure 1). During the early phase of PTI, after PRRs and LRR‐RK BAK1 form a receptor complex and bind to the ligand, RLCKs BIK1 or PBL1 are induced to phosphorylate respiratory burst oxidase homologue D (RBOHD) under flg22 induction. This process results in an intracellular oxygen burst that activates defence signalling (Figure 4) (Couto and Zipfel, 2016; Loo *et al*., 2022). This process is a critical signal not only for local immunity but also for cell‐to‐cell communication (Marcec *et al*., 2019). RIPK, a member of the RLCK VII family, is involved in regulating multi‐layered ROS production within the immune system. The *ripk* mutant produces less ROS when the immune response is triggered, making it susceptible to necrotic bacteria. RIPK can directly phosphorylate RBOHD to mediate ROS signalling (Li *et al*., 2021b). GDP‐L‐galactose phosphorylase (TaVTC2) modulates plant ascorbic acid (AsA) to mitigate oxidative stress induced by pathogen infection. Recent research has shown that when plants are infected with wheat mosaic virus (WYMV), TaVTC2 downregulates the enzyme activity, triggering a burst of ROS to resist WYMV (Tianye *et al*., 2023). The MAP4K family, activated by effectors during mid‐PTI, can not only phosphorylate the MAPK cascade but also transphosphorylate RLCKs; for instance, MAP4K SIK1 can bind, phosphorylate and stabilize BIK1, associating with phosphorylated RBOHD to enhance ROS production (Figure 4) (Zhang *et al*., 2018). Apart from this, the MAPK‐AL7 module as a negative regulator of ROS scavenging genes, promoting NLRs‐mediated immunity (Zhang *et al*., 2023a). Elevated intracellular calcium ions are also crucial for RBOHD activation, studies have found that Calcium‐dependent protein Kinases CPKs can also phosphorylate RBOHD to promote ROS production of during the immune response (Dubiella *et al*., 2013; Kadota *et al*., 2015; Wang *et al*., 2018a). While low concentrations of ROS serve as signalling molecules, excessive ROS can cause oxidative damage to lipids and proteins, prompting plants to tightly regulate ROS homeostasis to prevent cell damage (Jaspers and Kangasjarvi, 2010). ROD1 mutants confer broad‐spectrum resistance to plants against various bacterial and fungal pathogens.

As immunity progresses, ROD1 facilitates the breakdown of hydrogen peroxide by activating *CatB*, and its stability is governed by an E3 ubiquitin ligase. Interestingly, ROD1 has been found to share structural similarities with the fungal effector AvrPiz‐t, suggesting that it may utilize comparable mechanisms for protein degradation and ROS clearance (Gao *et al*., 2021). In the ETI pathway, which extends the MAPK cascade initiated during PTI, the Arabidopsis responds to effector‐induced surges of LCBs (long‐chain bases) and ceramides by triggering a bidirectional ROS production. Beyond the transient ROS generation by NADPH oxidase, the downstream MAPK6 associated with LCBs can act upstream to sustain ROS production, a process linked to PCD (Saucedo‐Garcia *et al*., 2023; Tsuda *et al*., 2013). Delving into the role of ROS signalling in plant immunity is pivotal for deepening our comprehension of the intricate mechanisms that underpin plant defence against pathogenic bacteria.

### Calcium ion signalling

**Figure 5:**
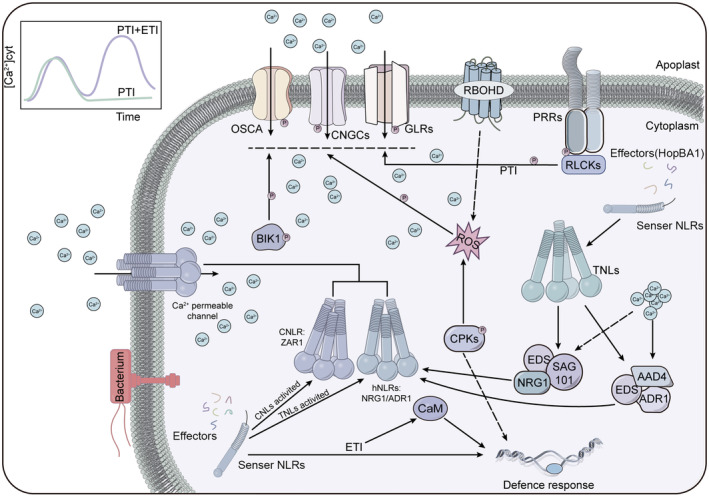
A proposed model of the Ca^2+^ signalling in plant immunity. Ca^2+^, as a secondary messenger, receives environmental stimuli to regulate cytoplasmic Ca^2+^ concentration and activate downstream signalling pathways. The recognition receptors PRRs and NLRs on the cell surface and inside perceive pathogens and activate PTI and ETI defence responses. Calcium ion channels OSCA, CNGCs and GLRs are phosphorylated by phosphokinases RLCKs and BIK1 to accelerate the transfer of extracellular calcium ions into the cell (Bjornson *et al*., 2021; Chin *et al*., 2013; Li *et al*., 2013; Thor *et al*., 2020; Tian *et al*., 2019; Upadhyay, 2022). CC‐NLRs ZAR1 and hNLRs NRG1 and ADR1 are induced by intracellular effectors to form Ca^2+^‐permeable channels before loss of membrane integrity and transfer to the plasma membrane, while TNLs tetramerize and rely on EDS1‐SAG101‐NRG1 or EDS1‐PAD4‐ADR1 transduces signals to Ca^2+^ channels (Adachi *et al*., 2019; Bi *et al*., 2021; Jacon *et al*., 2021; Wan *et al*., 2019; Wang *et al*., 2019; Yu *et al*., 2019; Zhao *et al*., 2021a). The ROS signal generated by RBOHD also leads to the accumulation of intracellular calcium ions, and CaM, and CPKs bind calcium ions to trigger defence responses (Dubiella *et al*., 2013). Arrows and end‐blocked lines indicate positive and negative regulation respectively. The letter P indicates phosphorylation.

Ca^2+^ serves as a universal secondary messenger across all eukaryotic organisms. Environmental stimuli prompt a swift rise in cytoplasmic Ca^2+^ concentration, which in turn activates downstream components (Figure 5). Alterations in intracellular Ca^2+^ levels are well‐documented downstream of PRR and NLR activation, with downstream signalling elements such as BIK1 and PBL1 being essential for Ca^2+^ signalling (Figures 1, 5) (Monaghan *et al*., 2014; Ranf *et al*., 2014). Recent findings in plant immunity have identified several plasma membrane‐localized Ca^2+^ channels, including CNGCs (Chin *et al*., 2013; Tian *et al*., 2019), OSCAs (Thor *et al*., 2020; Upadhyay, 2022) and GLRs (Bjornson *et al*., 2021; Li *et al*., 2013). CNGC2 and CNGC4 proteins form functional calcium channels that are blocked by calmodulin at rest. Upon pathogen attack, BIK1 is induced by ligand‐bound PRRs early in PTI to phosphorylate CNGC4, but not CNGC2, thereby activating calcium ion channels and leading to an increase in intracytoplasmic calcium ion concentration (Tian *et al*., 2019). The PRR FLS2 detects the PAMP flg22 and subsequently induces BIK1 to rapidly phosphorylate the Arabidopsis Ca^2+^ permeation channel OSCA1.3 within minutes, triggering Ca^2+^ influx and controlling stomatal closure (Thor *et al*., 2020). Furthermore, research has uncovered a new genetic component of the innate immune system in plant leaves, AtGLR3.3. GLR3.3 is a Ca^2+^ channel situated on the plasma membrane, which induces a transient increase in intracellular Ca^2+^ when exogenously applied GSH, and simultaneously significantly inhibits pathogens growth in a GLR3.3‐dependent manner (Li *et al*., 2013). Additionally, studies have shown that GLuRs, acting as Ca^2+^ channels on the plasma membrane, when overexpressed in *Arabidopsis thaliana*, can mediate jasmonic acid (JA) signal transduction, potentially enhancing resistance to necrotic fungi through both Ca^2+^ and JA signalling pathways (Kang *et al*., 2006).

Ca^2+^ transient signalling is pivotal in plant immunity. When challenged with *Pseudomonas syringae*, Arabidopsis exhibits two distinct Ca^2+^ signal peaks, with the second being stronger and more sustained, suggesting that PTI and ETI may elicit distinct Ca^2+^ signals (Koster *et al*., 2022). In PTI, a transient increase in cytoplasmic Ca^2+^ represents an early critical step, whereas ETI is chariacterized by a more robust and sustained influx of cytoplasmic Ca^2+^. Evidence suggests that Ca^2+^ permeable channel activity may be a common mechanism in plant NLR signalling, with elevated cytosolic Ca^2+^ concentration acting as a crucial signal for NLR to activate host defence responses (Wang *et al*., 2023a). In plants, regulated Ca^2+^ influx is also essential for the HR cell death associated with ETI (Moeder *et al*., 2019). The Arabidopsis recessive mutant *cngc20*, which exhibits intracytoplasmic Ca^2+^ accumulation and autoimmunity, is dependent on EDS1 and salicylic acid (SA), an early component of ETI. This mutant is sensitive to ROS, and accelerates host cell death in response to ETI (Zhao *et al*., 2021a,b). While Ca^2+^ serves as an upstream regulator in ETI and ROS pathways, the use of the calcium‐channel blocker LaCI3 has shown to inhibit the increase in cytoplasmic Ca^2+^, as well as the accumulation of H_2_O_2_ and HR hypersensitivity (Grant *et al*., 2000). In studies of plant resistance mechanisms, it has been noted that the activation of CNLs and TNLs pathways can influence the activity of Ca^2+^ permeation channels and mediate ETI responses (Jacon *et al*., 2021; Wang *et al*., 2023a). ZAR1, a typical CC‐RNL, acts as a sensor of pathogenic effectors, exhibits calcium‐permeable cation‐selective channel activity and is induced by AvrAC from *Xanthomonas campestris*, leading to elevated cytosolic Ca^2+^, triggering immunity and cell death (Bi *et al*., 2021). Upon recognition of intracellular effectors by sensor NLRs, the five N‐terminal α1‐helices of ZAR1 form a Ca^2+^‐permeable channel and translocate to the plasma membrane to facilitate Ca^2+^ influx. Additionally, hNLRs activated by TNLs also possess Ca^2+^‐permeable channel activity (Adachi *et al*., 2019; Jacon *et al*., 2021; Wang *et al*., 2019). Unlike CNLs, TNL resistosomes are NADasas. For example, TNLs recognize bacterial type III effector protein HopBA1 (RBA1), induce N‐terminal tetramerization of four TNLs, and rely on EDS1‐SAG101‐NRG1 or EDS1‐PAD4‐ADR1 to transduce signals to hNLRs Ca^2+^‐permeable channels, and the elevated intracellular Ca^2+^ concentration constitutively activates EDS1 (Wan *et al*., 2019; Yu *et al*., 2019; Zhao *et al*., 2021a,b).

## Conclusion and prospect

Cell sensor receptors integrate pathogen signals and orchestrate a cascade of reactions, progressively amplifying the plant's defence response, akin a domino effect that resonates throughout the organism. The immune system operates in a state of interdependent. For example, PTI can be seen as the precursor to ETI, with ETI acting as an intensified version of PTI, amplifying the signal and extending its duration. There is a dynamic interplay between transcription factors, as well as synergistic enhancement of signals, exemplified by the accumulation of ROS, which is regulated not only by PTI but also activated by Ca^2+^. Furthermore, there exists a counterbalancing antagonism among signals, such as the mutual antagonism between JA and SA pathways. Despite this, our understanding of the intricacies of plant immunity remains limited, and it remains to be established whether pathogens influence the crosstalk between these signalling pathways. These sophisticated mechanisms are central to the nuanced defence strategies of plants against a myriad of pathogens, and they also lay the groundwork and point the direction for future research endeavours.

## Conflict of interest

The authors declare no competing financial interests.

## Supporting information


**Figure S1** Working model of phosphorylation of RLCKs exemplified by BSK1 and BIK1.
**Figure S2** Simplified schematic representation of the MAPK cascade involved in the regulation of immunity by plant defence hormones.
