# Supplementary material for: PTI‐ETI synergistic signal mechanisms in plant immunity
Source: Plant Biotechnol J. 2024 Mar 12;22(8):2113–28. doi: 10.1111/pbi.14332 (PMC11258992; doi:10.1111/pbi.14332)
Supplement: Supplementary file 1 — Figure S1 Working model of phosphorylation of RLCKs exemplified by BSK1 and BIK1. Figure S2 Simplified schematic representation of the MAPK cascade involved in the regulation of immunity by plant defence hormones. [file PBI-22-2113-s001.docx]

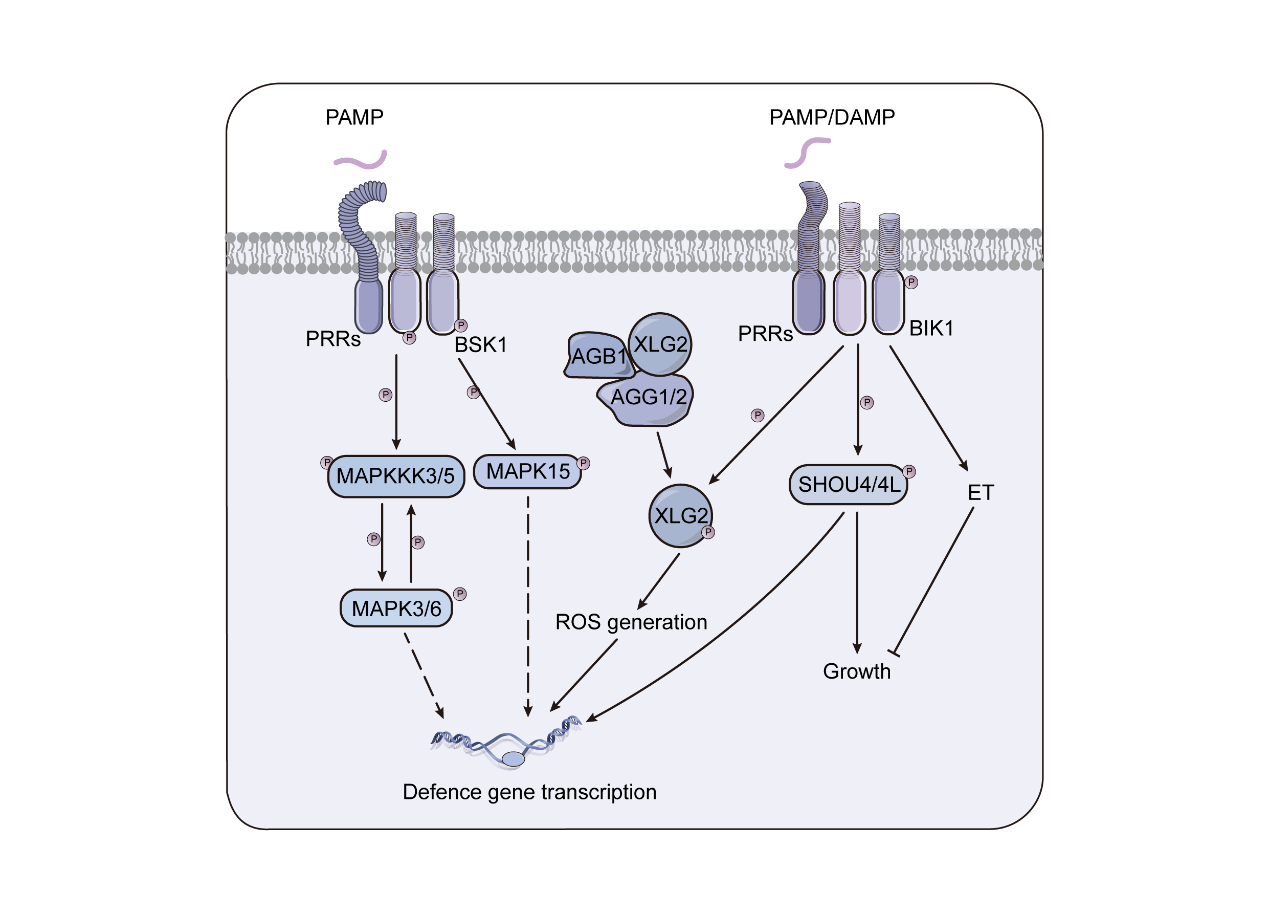


Supplementary Fig S1. Working model of phosphorylation of RLCKs exemplified by BSK1 and BIK1. The recognition of PAMPs or DAMPs by PRRs on the cell surface requires co-receptor RLCKs to assist in transmitting signals downstream, regulating MAPK cascades, ROS production, hormone synthesis regulation, growth, and development (Liang & Zhou, 2008). PRRs and BSK1 are induced by PAMP to trigger the phosphorylation of MAPKKK3/5, thereby activating MPK3/6 (Bi et al., 2018). MAPK15 can be directly phosphorylated by PAMP-stimulated enhanced BSK1, activating the transcription of downstream defense genes (Shi et al. al., 2022). XLG2, AGB1, and AGG1/2 regulate PAMP-triggered immunity by directly coupling to the FLS2 and BIK1 receptor complexes. XLG2 dissociates from AGB1 and is phosphorylated by BIK1, regulating downstream responses including ROS production (Bommert et al., 2013; Liang et al., 2016; Wang et al., 2016). In addition, BIK1 phosphorylation is involved in ET defense signaling (Liu et al., 2019). Induced by PAMP/DAMP, BIK1 directly phosphorylates SHOU4/4L and participates in defense regulation (Wang et al., 2023d). Arrows and end-blocked lines indicate positive and negative regulation, respectively. The letter P indicates phosphorylation.


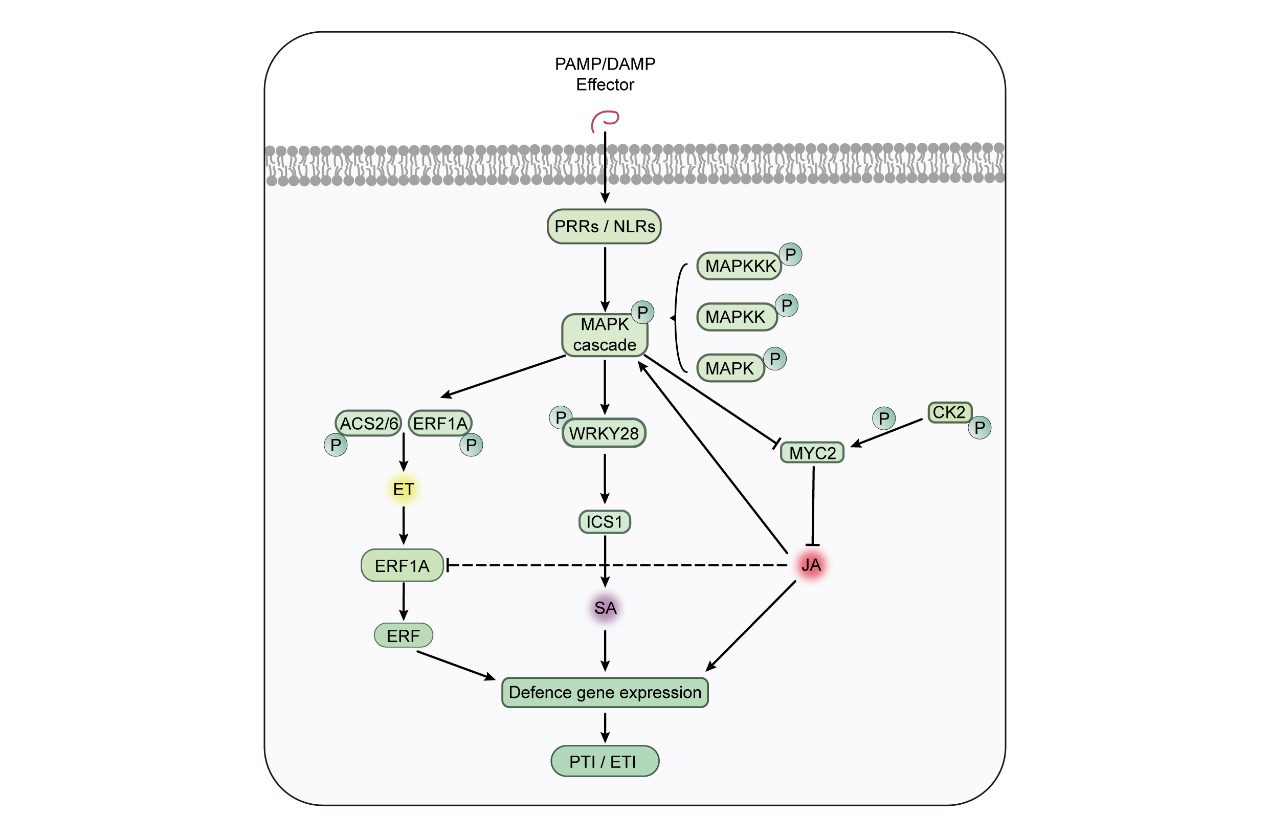


Supplementary Fig S2. Simplified schematic representation of the MAPK cascade involved in the regulation of immunity by plant defense hormones. Salicylic acid, jasmonic acid, and ethylene are the main defense phytohormones. Cells perceive pathogens to regulate the synthesis of salicylic acid, jasmonic acid, and ethylene. They can control the expression of genes related to plant hormone synthesis through the highly conserved MAPK cascade to induce downstream defense responses in the form of intercellular signaling. Arrows and end-blocked lines indicate positive and negative regulation, respectively. In the MAPK cascade, MKK3-MPK6 is activated by JA, and MKK3-MPK6 negatively regulates MYC2, a major transcription factor in the JA signaling pathway (Mine et al., 2017; Takahashi et al., 2007). As a positive regulator of JA, CK2 induces an increase in JA levels, and the kinase activity of CK2 phosphorylates MYC2, promotes the binding of MYC2 to the promoters of JA-related genes, and regulates JA signaling (Sun et al., 2023; Zhu et al., 2023). Ethylene synthesis and signal transduction are also controlled by MAPK cascades. The expression of ethylene synthesis-related genes ACS2 and ACS6 is enhanced by phosphorylation of the MAPK cascade, and the ethylene response factor ERF1A is also a substrate of the MAPK cascade (MPK3/MPK6). After being phosphorylated, it enhances the inhibition of ethylene biosynthesis and induces the expression of defense genes. (Broekaert et al., 2006; Meng & Zhang, 2013; Wang et al., 2022a). Regarding SA, the MAPK cascade directly phosphorylates WRKY28 after the PRR receptor is activated, and WRKY28 transcriptionally activates *ICS1* and participates in the biosynthesis and metabolism of SA (Navarro et al., 2004; van Verk et al., 2011). The letter P indicates phosphorylation.
